# Environmental Modulation of Perceived Cognitive Workload During a Memory Task: Evidence from Immersive Virtual Reality

**DOI:** 10.3390/jintelligence14080158

**Published:** 2026-08-01

**Authors:** Renato Orti, Michela Possenti, Luigi Lorenzo Luca Napolitano, Michela Romano, Sabrina Iuliano, Scila Nunziata, Angelo Lucio Silvino, Francesco Ruotolo

**Affiliations:** Laboratory of Cognitive Science and Immersive Virtual Reality (CS-IVR), Department of Psychology, University of Campania “L. Vanvitelli”, Viale Ellittico, 31, 81100 Caserta, Italy

**Keywords:** immersive virtual reality, urban parks, restorative environments, working memory, SIM-TLX, cognitive workload

## Abstract

Understanding how environmental contexts influence cognitive functioning is a key issue in cognitive science and applied ergonomics. This study examined whether everyday environmental settings modulate task performance and perceived cognitive workload during a card-matching memory task, using high-fidelity immersive Virtual Reality (VR) environments. Participants performed the task by sequentially uncovering cards and remembering their spatial locations to identify matching pairs in two virtual scenarios: a naturalistic environment (urban park) and an artificial setting (office). The number of moves and time of execution were collected as behavioral measures. Perceived cognitive workload was assessed after task completion in each condition using the Simulation Task Load Index (SIM-TLX). Behavioral performances did not differ between environments. However, participants reported lower perceived cognitive workload when performing the task in the naturalistic environment. This effect was not uniform across individuals: the reduction in perceived cognitive workload was primarily observed among participants exhibiting high levels of task efficiency. These findings suggest that environmental context interacts with individual cognitive efficiency by modulating the perceived cognitive workload without necessarily affecting objective performance outcomes. Overall, the results suggest that naturalistic immersive virtual contexts may mitigate perceived cognitive workload during memory-based tasks, highlighting the role of contextual factors in influencing cognitive efforts under ecologically valid conditions.

## 1. Introduction

Many everyday activities require remembering where objects are located while searching for the correct item (e.g., recalling where office or car keys were last left). Whether performing such tasks in a conventional office workspace or in a naturalistic outdoor environment leads to differences in cognitive efficiency remains an open question (Ohly et al., 2016).

Successfully performing cognitively demanding tasks requires individuals to allocate attention to task-relevant information while maintaining goal-directed behaviour over time. This process relies on controlled attentional mechanisms that maintain task-relevant representations and inhibit distraction (Baddeley, 1992; Engle, 2002). However, human cognitive resources are inherently limited, making sustained task performance increasingly effortful and vulnerable under prolonged executive demand (Kahneman, 1973). Interestingly, several theoretical accounts have suggested that certain environments may support the recovery of cognitive functioning after sustained mental effort. For example, the Stress Recovery Theory (SRT; Ulrich, 1983; Ulrich et al., 1991) emphasizes the immediate affective and physiological recovery triggered by exposure to natural stimuli, whereas the Attention Restoration Theory (ART; R. Kaplan & Kaplan, 1989; S. Kaplan, 1995; S. Kaplan & Berman, 2010) focuses on the cognitive benefits derived from the interaction with natural environments. Consistent with these theoretical accounts, behavioural studies have reported that even brief interactions with natural settings can improve performance on tasks requiring sustained attention and other demanding cognitive processes (Berto, 2005; Ohly et al., 2016; Stevenson et al., 2018).

However, a methodological distinction should be noted. Most existing studies have adopted pre–post exposure paradigms, assessing the restorative effects of nature primarily as a recovery mechanism following a sustained cognitive effort (Ohly et al., 2016; Stevenson et al., 2018). Only a limited number of studies have examined how natural elements influence cognition during task execution using exposure-during-task-performance (EDTP) paradigms (for a review, see Ohly et al., 2016). This approach shifts the focus from nature as a post hoc recovery context to nature as an online moderator of cognitive activity. For instance, studies by Berry et al. (2014, 2015) showed that exposure to natural scenes reduced participants’ impulsivity in a decision-making task. Li and Sullivan (2016) found that the presence of window views overlooking natural landscapes enhanced students’ attention, measured through a working memory test. Similarly, exposure to natural environments has also been associated with increased creative performance (Studente et al., 2016; Van Rompay & Jol, 2016).

More recently, the integration of Immersive Virtual Reality (IVR) has further advanced this line of research. By providing precise experimental control together with high levels of presence, that is the subjective sense of “being there” within a virtual environment (Sanchez-Vives & Slater, 2005), IVR allows researchers to isolate specific environmental features that may contribute to cognitive recovery or strain while maintaining ecological validity. Recent evidence suggests that virtual natural environments can reproduce some of the restorative benefits associated with physical nature (Chirico & Gaggioli, 2019), with studies reporting reductions in perceived cognitive workload and improvements in attentional engagement in digital workspaces (Yu et al., 2018; Mattila et al., 2020; Ruotolo et al., 2024). For example, Mattila et al. (2020) demonstrated that nature-based virtual experiences can foster restoration, providing a promising approach for mitigating cognitive fatigue in demanding work environments. However, it remains unclear whether naturalistic immersive contexts may affect cognitive effort during ongoing task performance.

A further methodological issue concerns the widespread reliance on behavioural task performance as the primary indicator of cognitive functioning. Although performance measures are commonly used to evaluate the cognitive effects of environmental exposure, they may fail to capture the mental effort required to sustain task engagement. In this regard, Hockey’s (1997) Compensatory Control Model provides a useful framework. According to this model, individuals may maintain stable task performance by increasing or decreasing the cognitive effort required to meet task goals. From this perspective, cognitive workload represents a crucial complementary dimension, reflecting the subjective experience associated with the deployment of attentional resources during task execution, even when behavioural outcomes remain unchanged (Pessiglione et al., 2025). Integrating this framework allows us to examine whether naturalistic and artificial environments differ not only in terms of objective performance, but also in the perceived effort required to sustain it.

Adopting an exposure-during-task-performance (EDTP) paradigm, the present study investigates whether immersion in biophilic virtual environments, compared with matched artificial virtual ones, influences both behavioural performance and perceived cognitive workload during a memory task. To address this question, participants performed a card-matching memory task in which they sequentially uncovered cards and attempted to remember their spatial locations in order to identify matching pairs. The task captures the dynamic integration of object identity (“what”) and spatial location (“where”) information while requiring continuous visual search and memory updating (Hollingworth & Henderson, 2002; Peterson et al., 2007; Postma et al., 2008). Such processes are central to many everyday activities that involve locating and retrieving objects within complex environments. Accordingly, the task can be considered a complex cognitive paradigm that engages multiple attentional and memory-related processes, providing an ecologically meaningful context in which to examine cognitive workload during task execution (Parsons, 2015).

In addition, perceived cognitive workload was assessed using the Simulation Task Load Index (SIM-TLX; Harris et al., 2020), an adaptation of the NASA-TLX (Hart & Staveland, 1988) specifically developed for simulation-based environments. This self-report instrument provides a multidimensional assessment of participants’ subjective workload experienced during task performance.

On the basis of the literature reviewed above, we hypothesized that immersion in biophilic virtual environments would influence behavioural performance and reduce perceived cognitive workload relative to artificial virtual settings. Specifically, we examined whether participants would report lower perceived cognitive workload and potentially improved task performance when performing the task in the natural environment compared with the artificial one.

## 2. Materials and Methods

### 2.1. Participants

Based on previous studies, a sample size of N ≥ 40 has been considered sufficient for virtual reality experiments employing within-subject designs (Yu et al., 2018; Ruotolo et al., 2024). Accordingly, fifty-one (51) participants (*F* = 38) aged 18–35 years (M_age_ = 22.58; SD_age_ = 2.43) were recruited from a university student population. All participants declared normal or corrected-to-normal vision and did not report any neurological disorder. All participants gave their consent to take part in the study. Participants recruitment and testing were in conformity with the relevant local Ethics Committee requirements that approved the study (Ethical Committee Approval Code: 12/2026), and with the 2013 Declaration of Helsinki. Finally, all participants were screened for prior Virtual Reality experience during recruitment. Only participants who had used a VR system at least once before the experiment and who did not habitually use VR for work or entertainment were enrolled, ensuring minimal familiarity with the medium while avoiding the inclusion of expert or habitual VR users.

### 2.2. Setting and Immersive Virtual Reality Devices

The experiment took place in a soundproof room of the Cognitive Science and Immersive Virtual Reality Laboratory (CS-IVR, Department of Psychology, University of Campania “L. Vanvitelli”, Caserta, Italy). The virtual reality equipment included the Unity Game Engine software v2021.2.21.f1 (Unity Technologies, San Francisco, CA, USA) and the Oculus Rift S (Oculus VR, Menlo Park, CA, USA) head mounted display (HMD) equipped with two Fast-switch LCD displays for stereoscopic depth (resolution of 1280 × 1440 pixels per eye; global resolution: 2560 × 1440), a 115° field of view, an 80 Hz refresh rate, and five built-in cameras enabling six-degree-of-freedom (6-DOF) positional tracking of the HMD. Motor responses were collected through two Oculus Touch motion-tracked controllers.

### 2.3. Virtual Environments

Two virtual environments representing “Artificial” and “Natural” conditions were modelled with the Unity Game Engine Software (Unity Technologies, San Francisco, CA, USA).

**Figure 1:**
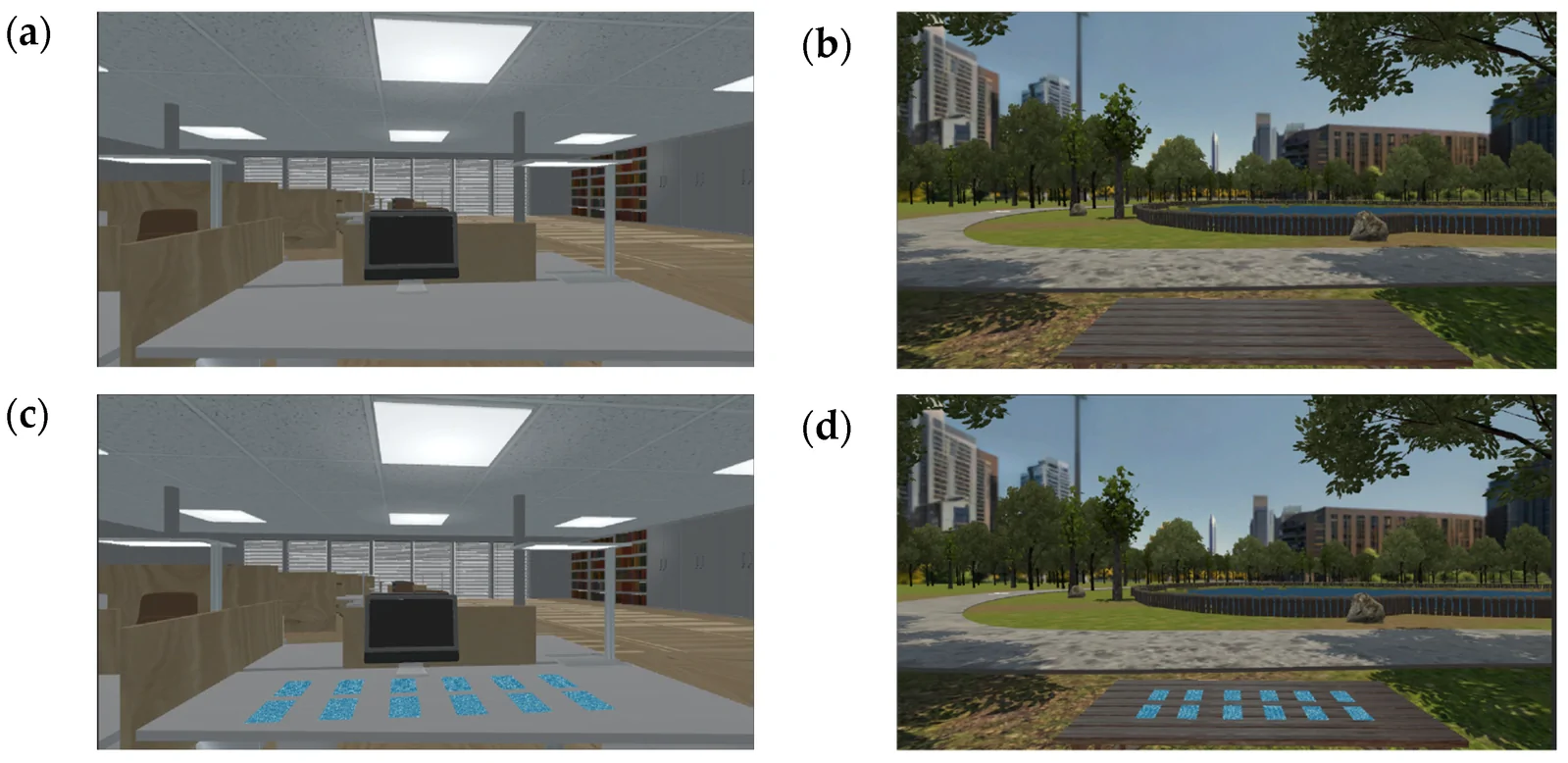
The figures depict the two virtual environments, namely the office (Artificial; (**a**,**c**)) and the urban park (Natural; (**b**,**d**)). Both virtual environments were modelled with the Unity Game Engine Software (Unity Technologies, San Francisco, CA, USA). (**a**,**b**) represent the two virtual environments without the cards of the card-memory matching task. (**c**,**d**) represent the two virtual environments with the array of cards placed on the desk (office) and picnic table (urban park) respectively.

The Artificial environment (Figure 1a) consisted of a simulated and multisensory open-plan office environment characterized by a neutral color palette and high spatial regularity. The setting featured multiple workstations equipped with standard computer hardware and ergonomic seating. A primary white desk, partitioned from the rest of the office, served as the immediate foreground and the participant’s workstation. Lighting was provided by a grid of recessed fluorescent-style LED panels, ensuring uniform illuminance across the work plane. The background included a library section with bookshelves and a glass-walled perimeter, suggesting a functional atmosphere. To complete the multisensory experience, a synchronized acoustic soundscape reproduced typical office noises, including ringing phones, running printers, keyboard typing, and ambient chatter.

The Natural environment (Figure 1b) depicted a multisensory urban park integrating natural and built elements. The foreground was centered on a wooden picnic-style table situated on a grass-covered surface, which served as the participant’s primary point of interaction. The midground was dominated by a pond enclosed by a low wooden perimeter fence and surrounded by deciduous trees. The background displayed an urban skyline. Correspondingly, the visual scene was paired with a natural soundscape featuring birds chirping and running water.

To ensure consistency across conditions, the participant’s viewpoint was fixed at a distance of approximately 0.3 m from the edge of the table or desk in both environments, maintaining a constant visual perspective.

### 2.4. Card-Matching Memory Task

The card-matching memory task integrated into both virtual environments consisted of twelve cards that were presented to the participants arranged in a 2 × 6 grid matrix (comprising two horizontal rows of six cards each; see Figure 1c,d).

The cards formed two sets of six identical pairs, representing stimuli semantically matched to the respective virtual environment (e.g., stone and flower for the Natural environment; printer and phone for the Artificial environment). This choice was intended to preserve semantic coherence between the target objects and their surrounding immersive context. An *a priori* computational analysis was performed through the RStudio software (v. 4.5.2), using the package *imager* (Barthelmé & Tschumperlé, 2019) to assess whether the two stimulus sets differed in low-level visual features. The sets were compared in terms of colour profile, luminance, and visual complexity, indexed through edge density. As expected, the sets differed in colour profile, t(9.87) = −2.81, *p* = 0.019, and luminance, t(9.99) = −3.24, *p* = 0.009, reflecting the intrinsic visual differences between natural and artificial items. Crucially, however, they did not differ significantly in visual complexity, t(6.24) = −0.87, *p* > 0.05, suggesting that structural complexity and shape-based processing demands were comparable across conditions.

The cards were positioned in a pseudo-randomized layout while maintaining a fixed distance from the participant to control for visual angle and reachability. In both conditions, the cards were placed on the horizontal surface in front of the participants (the white office desk in Figure 1c and the wooden picnic table in Figure 1d). This setup ensured that, although the surrounding environmental context varied (Artificial vs. Natural), the main cognitive task remained identical in terms of spatial configuration and task demands.

### 2.5. Questionnaire “Simulation Task Load Index (SIM-TLX)”

The Simulation Task Load Index (SIM-TLX) questionnaire (Harris et al., 2020) measures various aspects of mental workload in simulated environments using a 21-points Likert scale ranging from “low” to “high”. Participants rated their perceived workload on ten different subscales: *Mental Demands* (i.e., How mentally fatiguing was the task?), *Physical Demands* (i.e., How physically fatiguing was the task?), *Temporal Demands* (i.e., How hurried or rushed did you feel during the task?), *Frustration* (i.e., How insecure, discouraged, irritated, stressed, or annoyed did you feel?), *Task complexity* (i.e., How complex was the task?), *Situational Stress* (i.e., How stressed did you feel while performing the task?), *Distraction* (i.e., How distracting was the task environment?), *Perceptual Strain* (i.e., How uncomfortable or irritating were the visual and auditory aspects of the task?), *Task Control* (i.e., How difficult was the task to control?) and *Presence* (i.e., How present did you feel in the task?).

Participants completed the SIM-TLX questionnaire using the PsyToolkit web-based platform for research questionnaires (Stoet, 2017) after completing the task in each environmental condition. Consequently, the questionnaire was administered twice: once after completing the task in the office environment and once after completing the task in the urban park environment.

### 2.6. Procedure

Participants’ primary task was to identify the six identical pairs within the card array. No time constraints or limits on the number of attempts were imposed, allowing participants to complete the task at their own pace. For each trial, two main behavioral performance metrics were recorded: Number of Moves (calculated as the number of cards-pair inversions performed) and Time of Execution (defined as the latency between flipping the first card and identifying the final pair). In addition, a raw workload score from the SIM-TLX questionnaire (R-SIM-TLX) was calculated by aggregating the ratings across the ten subscales (Byers et al., 1989).

The order of presentation of the two environmental conditions was counterbalanced across participants to control for potential order effects. The total duration of the experimental session ranged between 10 and 15 min per participant.

### 2.7. Statistical Analysis

To evaluate possible order effects, preliminary analyses were conducted by treating the presentation order as a between-subjects factor. Participants were divided according to whether they completed the Artificial condition first or the Natural condition first. The two groups were compared on objective performance measures and self-reported workload dimensions through a series of independent-samples *t*-tests. No significant order-group differences emerged, indicating that presentation order did not systematically affect behavioral performance or subjective ratings.

To assess the impact of Artificial and Natural environments on memory performance and cognitive workload, a series of linear mixed models (LMMs) were defined (see Table A2 in Appendix B), via the software RStudio, using the *lme4* package (Bates et al., 2015).

Two LMMs were defined to assess the effect of environmental features on behavioral performance. In these models the Number of Moves (N Moves) and the Time of Execution (Time Ex) were defined as dependent variables, respectively. Participants entered into the model as a random intercept, whereas Condition (Artificial vs Natural) was treated as a fixed effect.

Ten LMMs (one for each SIM-TLX subscale) were defined to assess the effect of environmental features on the self-reported cognitive workload. In these models the participants’ scores on each SIM-TLX subscale were defined as dependent variables for each model. Again, participants entered into the models as random intercept, while Condition (Artificial, Natural) entered as a fixed effect. Moreover, in these models, Number of Moves (N Moves) and Time of Execution (Time Ex) were transformed into Z-scores and entered into the models as continuous predictors. The Z-score transformation of the continuous predictors (N Moves and Time Ex) facilitated the comparison of their effects and allowed for an intuitive interpretation: a value of 0 represented the sample mean (i.e., moderate task efficiency), while values of −1 and +1 represented one standard deviation below and above the average, respectively (i.e., high and low task efficiency). These behavioral metrics were included as continuous predictors to account for the potential influence of task performance on self-reported workload, thereby isolating the specific effect of the environmental condition on the perceived cognitive workload of each participant.

To control for Type-I error inflation due to multiple comparisons, *p*-values were corrected using the False Discovery Rate (FDR) method via the Benjamini–Hochberg procedure (Benjamini & Hochberg, 1995; Yekutieli & Benjamini, 1999). Following a homogeneous familywise error correction approach (Cramer et al., 2016), corrections were applied independently across the 10 subscales. Both unadjusted *p*-values and FDR-adjusted *p*-values (*p_adjusted_*) are reported.

To further characterize the sensitivity of the fitted LMMs given the available sample size, we conducted a Monte Carlo simulation-based sensitivity analysis using the *simr* package (Green & MacLeod, 2016). Because the three-way interactions represented the highest-order effects included in the models, the simulation-based analysis was restricted to these interaction terms. Power curves were estimated by varying the number of participants across predefined sample sizes and conducting 10,000 Monte Carlo simulations at each sample size. In each simulation, data were generated from the fitted models and the models were refitted to the simulated datasets to estimate power (1 − β), thereby assessing the probability of detecting the three-way interaction as a function of sample size.

## 3. Results

### 3.1. Behavioral Performance

Descriptive statistics of behavioral measures are reported in Table 1.

Neither of the two linear mixed models (LMMs) conducted on the number of moves and execution time showed a significant effect of condition (Artificial vs. Natural) (*F* < 1, *p* > 0.05). Overall, these results indicate that the environmental context did not significantly affect objective task performances.

### 3.2. Subjective Cognitive Workload (SIM-TLX)

#### 3.2.1. Mental Demands

**Figure 2:**
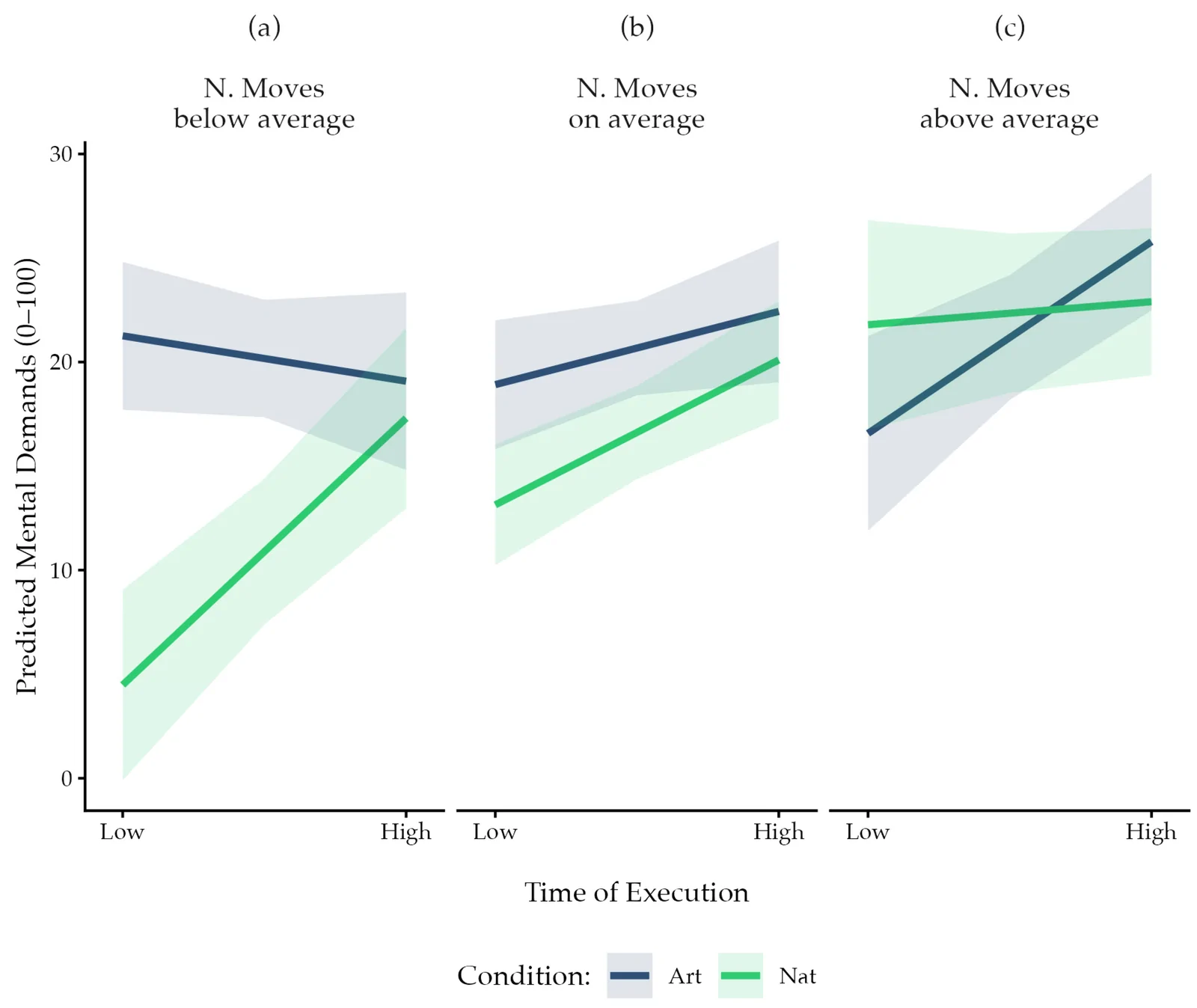
Interaction effects between Condition (Artificial vs. Natural), Number of Movements (N. Moves), and Time of Execution (Time Ex) on predicted Mental Demands. Panels represent participants with (**a**) high efficiency (below average moves), (**b**) moderate efficiency (on average moves), and (**c**) low efficiency (above average moves). Lines indicate estimated marginal means (EMMs), while shaded areas represent standard errors.

The LMM analysis of Mental Demands revealed a significant three-way interaction between Condition, Number of Movements, and Execution Time (*F*(1,67.96) = 7.47, *p* = 0.008, *p_adjusted_* = 0.040). Slope analyses showed that, for highly efficient participants, (below-average number of moves), the mental demands in the Natural condition were significantly lower than in the Artificial one at short execution times, but increased sharply as the time of execution increased (low time of execution: b = 6.41, β = 0.49, *p* = 0.02; mean time of execution: b = 3.48, β = 0.27, *p* = 0.05), converging toward the levels of the Artificial condition (see Figure 2a). Conversely, for less efficient participants (above-average number of moves), the Artificial condition showed a trend toward a more pronounced increase in mental demand as execution time increased (b = 4.61, β = 0.25, *p* = 0.09).

#### 3.2.2. Physical Demands

**Figure 3:**
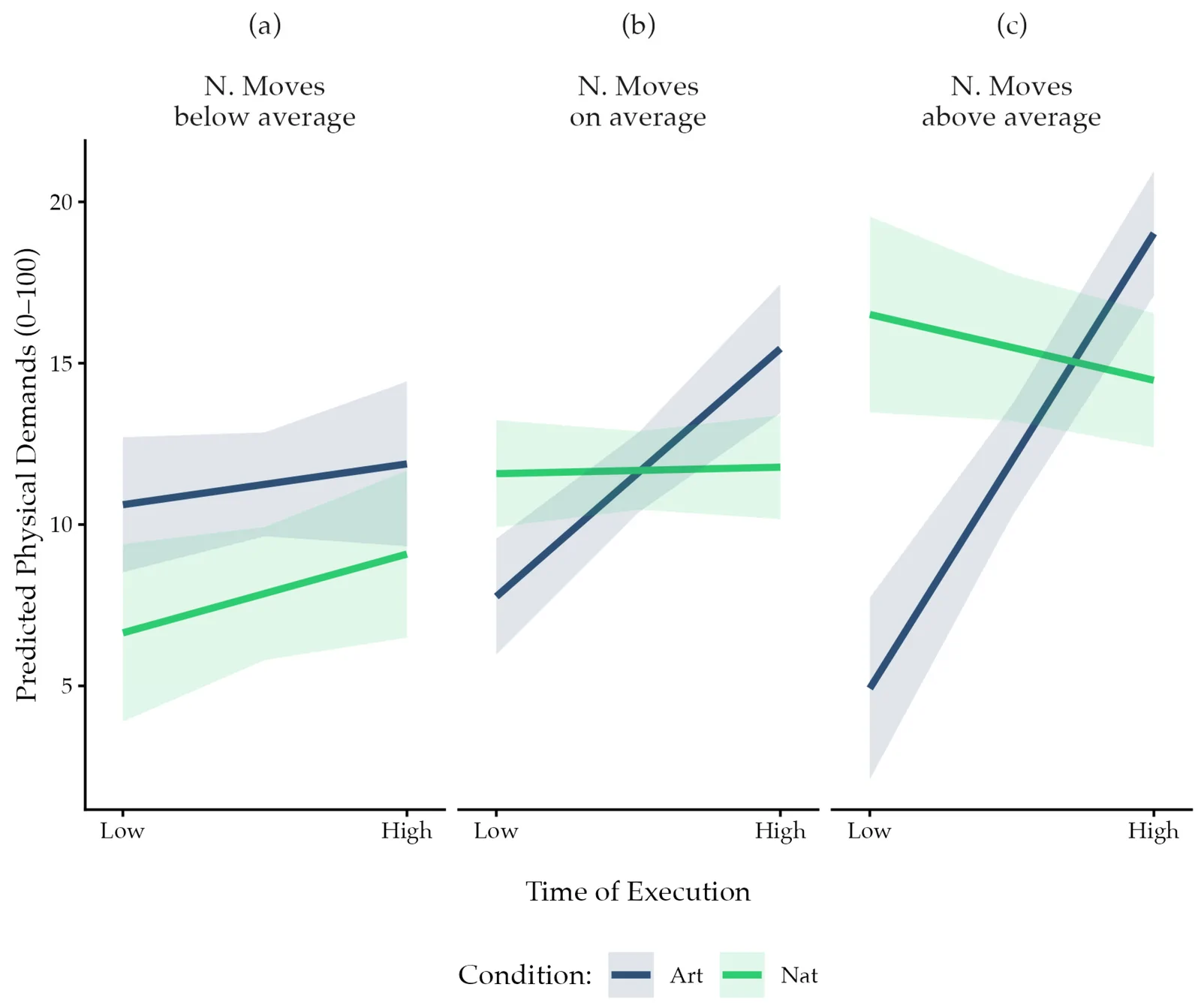
Interaction effects between Condition (Artificial vs. Natural), Number of Movements (N. Moves), and Time of Execution (Time Ex) on predicted Physical Demands. Panels represent participants with (**a**) high efficiency (below average moves), (**b**) moderate efficiency (on average moves), and (**c**) low efficiency (above average moves). Lines indicate estimated marginal means (EMMs), while shaded areas represent standard errors.

The LMM for Physical Demands also revealed a significant three-way interaction between Condition, Number of Movements, and Execution Time (*F*(1,80.16) = 11.01, *p* = 0.001, *p_adjusted_* = 0.014). Slope analyses showed that in the Artificial condition, perceived physical demands increased sharply with execution time, particularly when participants performed an average (b = 3.84, β = 0.32, *p* = 0.01) or a high number of moves (b = 7.05, β = 0.59, *p* < 0.01; see Figure 3b,c). Notably, this trend was absent or reversed in the Natural condition, where physical demand remained relatively stable despite longer execution times.

#### 3.2.3. Task Control

The LMM for Task Control revealed a significant effect of the Time of Execution (*F*(1,87.50) = 8.30, *p* = 0.005, *p_adjusted_* = 0.05), with participants reporting higher Task Control proportional to the time spent to complete the task (b = 3.52, β = 0.24, *p* = 0.006).

#### 3.2.4. Temporal Demands and Frustration

Regarding Temporal Demands and Frustration, the models revealed tendent main effects of the Number of Movements (*F*(1,73.40) = 7.18, *p* = 0.009; *p_adjusted_* = 0.079; *F*(1,68.77) = 6.12, *p* = 0.016, *p_adjusted_* = 0.079 respectively). Specifically, both dimensions increased proportionally with the number of movements required to complete the task (Temporal demands: b = 7.49, β = 0.31, *p* = 0.01; Frustration: b = 3.72, β = 0.25, *p* = 0.02).

#### 3.2.5. Task Complexity, Situational Stress, Distraction, Perceptual Strain and Presence

No statistically significant predictors emerged for Task Complexity, Situational Stress, Distraction, Perceptual Strain, or Presence. Detailed statistics for these models are provided in Appendix B.

Overall, these results suggest that the environmental context did not directly influence objective task performance, but interacted with it in shaping specific dimensions of perceived cognitive workload.

### 3.3. Monte Carlo Simulation-Based Sensitivity Analysis

The simulation-based power curves indicated that, at the observed sample size (N = 51), the estimated power to detect the three-way interaction was 76.21% (95% CI = 75.36–77.04) for the Mental Demands model and 89.97% (95% CI = 89.36–90.55) for the Physical Demands model.

## 4. Discussion

The present study aimed to investigate the influence of natural compared to artificial virtual environments on behavioral performance and perceived cognitive workload during a card-matching memory task. Our primary objective was to move beyond the traditional post hoc recovery paradigms to examine the possible online influence of nearby natural environmental contexts, such as urban parks. By integrating immersive virtual reality with multidimensional measures of perceived cognitive workload, we sought to capture the asymmetries between objective performance and the subjective effort of task engagement. Overall, while the environmental context did not modify the objective efficiency of task completion, it modulated specific dimensions of perceived cognitive workload, revealing an interaction between an individual’s task number of moves, execution time, and environmental conditions.

### 4.1. Behavioural Performance

Contrary to our expectations, behavioral performance did not differ significantly between the Natural and Artificial conditions. Participants completed the task with comparable numbers of moves and similar execution times in both environments. In this regard, the absence of direct behavioral effects from natural environments is becoming a recurrent finding in the recent literature (see Fu et al., 2025 for a review). However, rather than suggesting a lack of environmental impact, our findings suggest that stable behavioral outcomes may conceal differences in the cognitive resources recruited to sustain task performance across different environmental contexts. This sounds like proof if considering that behavioral measures may be susceptible to limited sensitivity in capturing subtle variations in cognitive processing. Indeed, as argued by Pessiglione et al. (2025), performance often remains stable despite the internal use of compensatory strategies (see also Hockey, 1997).

### 4.2. Perceived Cognitive Workload

Notably, the evaluation of the subjective cognitive workload revealed differences in specific dimensions of perceived workload that behavioral metrics failed to capture. In particular, the analysis of the SIM-TLX subscales revealed significant effects of the context especially on mental and physical efforts.

The analysis of Mental Demands showed that participants reported less mental fatigue in the Natural compared to the Artificial condition but only when participants performed the task efficiently, namely with a low number of moves and in a rapid to average time of execution. These findings suggest that while natural elements facilitate the recovery of depleted resources and may support attentional functioning (Ohly et al., 2016), their benefits also appear to be contingent upon the individual performance threshold. Specifically, when execution is prolonged or less efficient, the top-down cognitive cost required to sustain goal-directed attention may supersede the restorative effects of the environment (S. Kaplan, 1995).

While the modulation of mental demand appeared to be sensitive to individual efficiency and task duration, a distinct pattern emerged for Physical Demands. In this dimension, the natural environment appeared to attenuate the increase in perceived physical effort, decoupled from the performance-related fluctuations observed in Mental Demands. Specifically, while the Artificial condition was characterized by a sharp increase in perceived physical exertion as execution time and number of moves increased, the Natural condition showed relatively stable levels of perceived physical effort across execution times. This pattern may be interpreted in light of the Stress Recovery Theory (SRT), which suggests that natural stimuli are often associated with physiological relaxation responses (Ulrich et al., 1991). In the Artificial condition, the accumulation of task-related moves and longer execution times may have increased the subjective awareness of physical strain. Conversely, the natural environment may have functioned as a hedonic distractor (i.e., soft fascination; R. Kaplan & Kaplan, 1989), potentially attenuating the perception of fatigue (Berto, 2014). This interpretation is consistent with previous evidence suggesting that nature-based contexts can mitigate the perceived strain of physical tasks, even when the mechanical demands remain constant (Ryan et al., 2010).

Despite the broader effects of environmental context on mental and physical demands, certain dimensions of cognitive workload appeared to be more sensitive to the mechanics of the task. Particularly, the analysis of Task Control revealed a significant positive correlation with execution time. Participants reported a higher sense of control as the duration of the task increased. This pattern may reflect a gradual familiarization with the task structure within both immersive environments. In this sense, longer execution times may allow participants to adopt a more deliberate pace, which in turn may enhance the perceived sense of control over task performance.

Conversely, the results regarding Temporal Demands and Frustration revealed, in tendency, a linear relationship with the number of moves executed to complete the task. Therefore, as the efficiency in finding the card-matching sequence decreased (i.e., more movements required), participants reported a proportional increase in time pressure and frustration. From an applied perspective, this suggests that while biophilic virtual environments might act as a cognitive buffer, they cannot fully compensate for a fundamentally demanding task.

Finally, the lack of significant differences in Task Complexity, Situational Stress, Distraction, Perceptual Strain, and Presence across conditions is particularly telling. Specifically, the absence of significant differences in Task Complexity, Situational Stress, Distraction, and Perceptual Strain supports the methodological comparability of the two immersive environments. These dimensions are particularly relevant in VR settings, as they may capture simulation-induced strain, distracting environmental features or frustration generated by the virtual setup itself. If one environment had produced higher scores on these dimensions, differences in perceived cognitive workload could have been attributed to a less comfortable simulation. Instead, their stability across conditions suggests that the two environments were comparable in terms of general discomfort and distracting features. Coherently, the lack of differences in Presence across conditions suggests that the observed effects were unlikely to be driven by differences in immersion: indeed, participants reported an equivalent sense of being “there” in both virtual environments, yet they processed the subjective cost of their efforts in different ways.

Taken together, our findings uncover a crucial decoupling effect: while objective behavioral performance remains stable across settings, the perceived cognitive load required to sustain that performance fluctuates dynamically depending on the interaction between the environment, number of moves and execution time. Such a conditional pattern can be interpreted according to Hockey’s (1997) Compensatory Control Model. The absence of significant differences in objective performance suggests that participants were able to maintain comparable task outcomes across environments. However, the differences observed in perceived cognitive workload indicate that the subjective cost of maintaining performance varied as a function of environmental context and behavioral efficiency. In this sense, the naturalistic environment may have reduced the perceived regulatory cost of task execution, particularly when participants’ baseline efficiency allowed them to benefit from the environmental context. Conversely, when task execution became less efficient, the cognitive effort required to maintain performance may have outweighed the potential perceived cognitive workload reducing effect of the naturalistic environment.

According to this framework, human performance is maintained through a self-regulatory loop that compensates for increased task demands or environmental stressors by mobilizing additional mental effort (Pessiglione et al., 2025). In our study, the Artificial environment appeared to function as a contextual stressor that increased the regulatory overhead required to maintain performance standards. Participants achieved the same level of accuracy as in the Natural condition, but reporting a higher perceived cognitive workload, as evidenced by the elevated Mental Demands and Physical demand scores. Conversely, the Natural context might have allowed the cognitive system to maintain high performance while operating in a lower-effort state.

Taken together, these results seem to suggest that biophilic contexts do not merely offer aesthetic pleasure but may function as cognitive buffers that reduce the effort-to-performance ratio. However, this interpretation should be considered provisional and requires further investigation.

Finally, although a fully naturalistic setting would likely have maximized the contrast between artificial and natural conditions, the present study aimed to examine a form of nature exposure that is commonly embedded in everyday urban contexts (i.e., urban parks). Such a solution provided an ecologically valid and practically relevant context for investigating how accessible green spaces may modulate perceived cognitive workload during ongoing task performance.

### 4.3. Limitations and Future Directions

Although the present study provides evidence that a naturalistic VR environment can buffer perceived cognitive workload without altering objective performance, few limitations should be noted.

While the demographic composition of the sample, primarily consisting of young adult university students with a prevalent female representation, may limit immediate generalizability to older cohorts or distinct occupational sectors, this homogeneity served as an advantageous methodological control. Indeed, by targeting this specific group, we effectively minimized confounding variables related to age-dependent cognitive variations or heterogeneous digital literacy, which was crucial for establishing a clean baseline and a reliable initial exploration of the EDTP paradigm in immersive VR. However, future research should validate and extend these findings across more diverse and representative populations.

Another limitation concerns the relatively modest sample size, which may have reduced the sensitivity to detect small or more complex effects. To further characterize the sensitivity of the present design, we conducted a Monte Carlo simulation-based sensitivity analysis. The resulting power curves estimated the probability of detecting the three-way interaction across a range of sample sizes. Although these analyses provide additional information about the sensitivity of the fitted models, replication in larger and more heterogeneous samples is needed to strengthen the generalizability of the present findings.

Additionally, despite measuring cognitive workload through a validated self-report tool (i.e., SIM-TLX), the integration of objective neurophysiological metrics, such as heart rate variability or pupillometry, would offer a broader and more nuanced picture of the actual cognitive demand. In this way, combining subjective, behavioral, and physiological measures would provide a more comprehensive understanding of how immersive virtual environments modulate cognitive workload during task execution.

Finally, while the present study focused on the use of only environment-congruent stimulus sets, this methodological choice opens an interesting avenue for future research. First, this choice aligned with the ecological rationale of the study. Second, preserving semantic coherence between the stimuli and the immersive scenarios was intended to avoid introducing context-incongruent objects that could have produced additional semantic interference during the task. This is theoretically consistent with evidence showing that objects are typically processed within meaningful contexts (Ruggiero et al., 2026), where contextual knowledge constrains which objects are expected and where they are likely to appear (Bar, 2004). It is also consistent with the broader use of immersive VR to study cognitive processes under conditions that combine experimental control with ecological validity (Garcia-Navarra et al., 2025; Ruotolo et al., 2026). Future studies may extend this design by orthogonally manipulating the environmental context and stimulus type, for example by comparing environment-congruent, environment-incongruent, and abstract stimuli.

## 5. Conclusions

Overall, the present study highlights the interaction between biophilic virtual environments, perceived cognitive workload and individual efficiency. The results suggest that virtual nature benefits do not uniformly translate into improved performance but rather depend on the individual’s efficiency during task execution.

These findings contribute to cognitive science research by clarifying how environmental context may influence the perceived cognitive workload without necessarily altering objective performance outcomes. From this perspective, biophilic virtual environments may represent a promising avenue for the design of personalized interfaces that take into account individual cognitive profiles and baseline efficiency.

From an applied ergonomics perspective, these findings suggest that environmental design may play a role not only in supporting performance but also in modulating the cognitive effort required to sustain it. Indeed, although the present findings were obtained in immersive VR, they may provide a useful basis for generating hypotheses about physical environments. VR allows environmental features to be experimentally controlled while preserving ecological characteristics that are difficult to reproduce in standard laboratory tasks. Nevertheless, direct translation to real-world settings should be made cautiously, as physical environments include additional sensory, social, and contextual variables.

In conclusion, integrating restorative environmental features into digital workspaces could therefore contribute to promoting a more sustainable balance between efficiency and psychological well-being.

## Figures and Tables

**Table 1:** Reporting the mean Number of Moves and Time of Execution (in seconds) across participants in the Artificial and Natural conditions.

Condition	Number of Moves Mean (SD)	Time of Execution Mean (SD)
Artificial	10.94 (2.19)	47.59 (22.65)
Natural	10.88 (1.28)	52.29 (30.18)

## Data Availability

The data that support the findings of this study are available from the corresponding author upon reasonable request.

## Abbreviations

The following abbreviations are used in this manuscript:

EDTP	Exposure During Task Performance
EMMs	Estimated Marginal Means
LMM	Linear Mixed-effects Model
ART	Attention Restoration Theory
SRT	Stress Recovery Theory

## Appendix A. Linear Mixed-Effects Models

**Table A1:** Schematic overview of the Linear Mixed-Effects Models (LMMs) carried out for the study. The table delineates the outcome (i.e., dependent) variables, fixed and random effects, and the corresponding formal specifications using the lme4 package syntax. Models 1–2 evaluated the impact of environmental conditions on behavioral performance, while Models 3–12 evaluated the multidimensional subjective workload (SIM-TLX) by examining the interactions between condition, time execution, and number of moves. In all models, participants (ID) were treated as a random intercept. The models were specified as: Dependent Variable ∼ Fixed Factor * Fixed Covariate (when applicable) + (1∣ID), where ~ denotes the model response function, * represents the main effects and their interaction, + adds a fixed covariate, and (1|ID) includes a random intercept per participant to account for repeated measures.

LMMs	Outcome	Fixed Factor(s)	Random Factor(s)	Formula (lme4 Syntax)
Model 1 (*lmm_behav_1*)	N Moves	Condition	Participants	N Moves ~ Condition + (1|ID)
Model 2 (*lmm_behav_2*)	Time Ex	Condition	Participants	Time Ex ~ Condition + (1|ID)
Model 3 (*lmm_selfrep_1*)	Mental Demands	Condition N Moves Time Ex	Participants	Mental Demands ~ Condition * N Moves * Time Ex + (1|ID)
Model 4 (*lmm_selfrep_2*)	Physical Demands	Condition N Moves Time Ex	Participants	Physical Demands ~ Condition * N Moves * Time Ex + (1|ID)
Model 5 (*lmm_selfrep_3*)	Temporal Demands	Condition N Moves Time Ex	Participants	Temporal Demands ~ Condition * N Moves * Time Ex + (1|ID)
Model 6 (*lmm_selfrep_4*)	Frustration	Condition N Moves Time Ex	Participants	Frustration ~ Condition * N Moves * Time Ex + (1|ID)
Model 7 (*lmm_selfrep_5*)	Task Complexity	Condition N Moves Time Ex	Participants	Task Complexity ~ Condition * N Moves * Time Ex + (1|ID)
Model 8 (*lmm_selfrep_6*)	Situational Stress	Condition N Moves Time Ex	Participants	Situational Stress ~ Condition * N Moves * Time Ex + (1|ID)
Model 9 (*lmm_selfrep_7*)	Distraction	Condition N Moves Time Ex	Participants	Distraction ~ Condition * N Moves * Time Ex + (1|ID)
Model 10 (*lmm_selfrep_8*)	Perceptual Strain	Condition N Moves Time Ex	Participants	Perceptual Strain ~ Condition * N Moves * Time Ex + (1|ID)
Model 11 (*lmm_selfrep_9*)	Task Control	Condition N Moves Time Ex	Participants	Task Control ~ Condition * N Moves * Time Ex + (1|ID)
Model 12 (*lmm_selfrep_10*)	Presence	Condition N Moves Time Ex	Participants	Presence ~ Condition * N Moves * Time Ex + (1|ID)

## Appendix B. Subjective Cognitive Workload (SIM-TLX) Additional Results

### Appendix B.1. Mental Demands

**Table A2:** LMM on Mental Demands—Omnibus ANOVA test. In bold are reported statistically significant results after FDR correction.

Parameter	df Num	df Den	*F*	*p*	*p_adjusted_*
Condition	1	46.98	3.63	0.063	0.244
N Moves	1	68.63	3.42	0.069	0.118
Time Ex	1	70.05	3.24	0.076	0.254
Condition × N Moves	1	69.95	2.36	0.129	0.644
Condition × Time Ex	1	68.07	0.36	0.550	0.611
N Moves × Time Ex	1	71.21	0.00	0.971	0.971
**Condition × N Moves × Time Ex**	**1**	**67.96**	**7.47**	**0.008**	**0.040**

**Table A3:** Slope analysis for the interaction between Condition and Number of Movements (N Moves) on Mental Demands. Asterisk denotes standardized values (i.e., standardized Beta value).

Condition	N Moves	b	β *	SE	*p*
Artificial	Below average	−1.09	−0.06	0.146	0.69
**Natural**	**Below average**	**6.41**	**0.49**	**0.21**	**0.02**
Artificial	On average	−1.76	−0.09	0.13	0.45
**Natural**	**On average**	**3.48**	**0.27**	**0.14**	**0.05**
**Artificial**	**Above average**	**4.61**	**0.25**	**0.15**	**0.09**
Natural	Above average	0.55	0.04	0.16	0.79

### Appendix B.2. Physical Demands

**Table A4:** LMM on Physical Demands—Omnibus ANOVA test. In bold are reported statistically significant results FDR correction.

Parameter	df Num	df Den	*F*	*p*	*p_adjusted_*
Condition	1	45.94	0.00	0.962	0.962
N Moves	1	81.07	4.04	0.048	0.118
Time Ex	1	82.96	4.87	0.030	0.151
Condition × N Moves	1	82.62	2.73	0.102	0.644
Condition × Time Ex	1	80.35	4.47	0.038	0.377
N Moves × Time Ex	1	83.90	2.50	0.118	0.968
**Condition × N Moves × Time Ex**	**1**	**80.16**	**11.01**	**0.001**	**0.014**

**Table A5:** Slope analysis for the interaction between Condition and Number of Movements (N Moves) on Physical Demands. Asterisk denotes standardized values (i.e., standardized Beta value).

Condition	N Moves	b	β *	SE	*p*
Artificial	Below average	0.63	0.05	0.14	0.71
Natural	Below average	1.22	0.17	0.23	0.47
**Artificial**	**On average**	**3.84**	**0.32**	**0.12**	**0.01**
Natural	On average	0.10	0.01	0.15	0.93
**Artificial**	**Above average**	**7.05**	**0.59**	**0.14**	**0.00**
Natural	Above average	−1.02	−0.14	0.17	0.42

### Appendix B.3. Temporal Demands and Frustration

**Table A6:** LMM on Temporal Demands—Omnibus ANOVA test.

Parameter	df Num	df Den	*F*	*p*	*p_adjusted_*
Condition	1	46.19	0.08	0.783	0.870
N Moves	1	73.40	7.18	0.009	0.079
Time Ex	1	75.11	0.00	0.965	0.965
Condition × N Moves	1	74.89	1.29	0.259	0.863
Condition × Time Ex	1	72.72	0.44	0.508	0.611
N Moves × Time Ex	1	76.26	0.68	0.412	0.968
Condition × N Moves × Time Ex	1	72.59	2.21	0.141	0.243

**Table A7:** LMM on Frustration—Omnibus ANOVA test.

Parameter	df Num	df Den	*F*	*p*	*p_adjusted_*
Condition	1	46.63	1.39	0.244	0.406
N Moves	1	68.77	6.12	0.016	0.079
Time Ex	1	70.23	0.63	0.431	0.825
Condition × N Moves	1	70.11	0.59	0.443	0.886
Condition × Time Ex	1	68.19	2.33	0.132	0.516
N Moves × Time Ex	1	71.39	1.46	0.230	0.968
Condition × N Moves × Time Ex	1	68.09	2.73	0.103	0.243

### Appendix B.4. Task Control

**Table A8:** LMM on Task Control—Omnibus ANOVA test. In bold are reported statistically significant results FDR correction.

Parameter	df Num	df Den	*F*	*p*	*p_adjusted_*
Condition	1	48.42	0.14	0.713	0.870
N Moves	1	85.84	0.77	0.381	0.424
**Time Ex**	**1**	**87.50**	**8.30**	**0.005**	**0.050**
Condition × N Moves	1	87.16	0.03	0.868	0.989
Condition × Time Ex	1	85.22	1.30	0.258	0.516
N Moves × Time Ex	1	88.18	0.26	0.609	0.971
Condition × N Moves × Time Ex	1	85.03	0.60	0.442	0.632

### Appendix B.5. Task Complexity Situational Stress, Distraction, Perceptual Strain and Presence

**Table A9:** LMM on Task Complexity—Omnibus ANOVA test.

Parameter	df Num	df Den	*F*	*p*	*p_adjusted_*
Condition	1	46.36	5.77	0.020	0.204
N Moves	1	59.24	4.47	0.039	0.118
Time Ex	1	60.10	0.06	0.815	0.906
Condition × N Moves	1	60.12	0.01	0.932	0.989
Condition × Time Ex	1	58.89	0.53	0.468	0.611
N Moves × Time Ex	1	61.05	0.01	0.940	0.971
Condition × N Moves × Time Ex	1	58.81	0.02	0.875	0.875

**Table A10:** LMM on Situational Stress—Omnibus ANOVA test.

Parameter	df Num	df Den	*F*	*p*	*p_adjusted_*
Condition	1	47.71	1.40	0.243	0.406
N Moves	1	67.06	3.36	0.071	0.118
Time Ex	1	68.33	0.29	0.593	0.825
Condition × N Moves	1	68.26	0.11	0.741	0.989
Condition × Time Ex	1	66.55	1.84	0.179	0.516
N Moves × Time Ex	1	69.45	0.84	0.363	0.968
Condition × N Moves × Time Ex	1	66.45	2.17	0.146	0.243

**Table A11:** LMM on Distraction—Omnibus ANOVA test.

Parameter	df Num	df Den	*F*	*p*	*p_adjusted_*
Condition	1	49.60	0.12	0.730	0.870
N Moves	1	85.39	1.04	0.311	0.388
Time Ex	1	87.02	0.19	0.660	0.825
Condition × N Moves	1	86.69	0.00	0.989	0.989
Condition × Time Ex	1	84.78	0.52	0.475	0.611
N Moves × Time Ex	1	87.71	0.49	0.484	0.968
Condition × N Moves × Time Ex	1	84.60	3.51	0.064	0.214

**Table A12:** LMM on Perceptual Strain—Omnibus ANOVA test.

Parameter	df Num	df Den	*F*	*p*	*p_adjusted_*
Condition	1	47.69	3.35	0.073	0.244
N Moves	1	84.70	0.42	0.519	0.519
Time Ex	1	86.44	0.22	0.642	0.825
Condition × N Moves	1	86.09	0.32	0.573	0.955
Condition × Time Ex	1	84.05	0.01	0.940	0.940
N Moves × Time Ex	1	87.19	0.02	0.900	0.971
Condition × N Moves × Time Ex	1	83.85	0.13	0.716	0.875

**Table A13:** LMM on Presence—Omnibus ANOVA test.

Parameter	df Num	df Den	*F*	*p*	*p_adjusted_*
Condition	1	45.32	2.71	0.107	0.267
N Moves	1	65.32	1.11	0.295	0.388
Time Ex	1	66.67	2.69	0.106	0.265
Condition × N Moves	1	66.59	0.62	0.435	0.886
Condition × Time Ex	1	64.79	1.47	0.230	0.516
N Moves × Time Ex	1	67.84	0.01	0.918	0.971
Condition × N Moves × Time Ex	1	64.68	0.05	0.830	0.875
